# Refractory Pit1 plurihormonal tumours and thyrotroph adenomas

**DOI:** 10.1007/s11102-023-01312-9

**Published:** 2023-04-29

**Authors:** Lenders NF, McCormack AI

**Affiliations:** 1 Department of Endocrinology, St Vincent’s Hospital, Sydney, NSW Australia; 2 Garvan Institute of Medical Research, Sydney, NSW Australia; 3 St Vincent’s Clinical School, University of New South Wales, Sydney, NSW Australia

**Keywords:** Pituitary tumours, Pit1 plurihormonal tumours, Thyrotroph adenomas, Pituitary adenomas

## Abstract

Pit-1 tumours are derived from neoplastic cells of either somatotroph, lactotroph or thyrotroph cell lineages, but there are also distinct mixed tumours and plurihormonal tumours within this category as described within the 2022 edition of the WHO classification of pituitary tumours. Plurihormonal tumours and thyrotroph adenomas are transcriptionally similar and grouped together to discuss in this review, although it is clear an immature type of plurihormonal tumour exists which are more commonly associated with refractory disease. Management of residual or recurrent disease should follow that of other aggressive pituitary tumours, although a trial of somatostatin analogue therapy is certainly warranted before considering temozolomide therapy.

## Introduction

The Pit-1 lineage of pituitary tumours has evolved in the 2022 WHO classification to encompass tumours derived not only from mature somatotroph, lactotroph and thyrotroph cells, but recognising distinct plurihormonal types and tumours originating from precursor cells (Table 1) [1]. From a prognostic standpoint, refining the classification of Pit-1 lineage tumours is important as type behaviour varies.

**Table 1:** The 2022 WHO classification of PIT1 lineage tumours. (adapted from Asa et al., 2022)

Type	Subtype	Hormones	Other features
Lactotroph	Sparsely granulated Densely granulated	PRL (paranuclear) PRL (diffuse)	Weak or negative LMWK
Somatotroph	Sparsely granulated Densely granulated	GH GH	Perinuclear LMWK Fibrous bodies > 70%
Mammosomatotroph #		GH (predominant), PRL, $\alpha$-subunit	Perinuclear LMWK
Mixed somatotroph and lactotroph #		Scattered GH and PRL	
Thyrotroph		$\alpha$-subunit, $\beta$TSH,	Weak or negative LMWK
Mature plurihormonal PIT-1 lineage *		Monomorphic tumour cells; predominant GH with variable PRL, $\beta$TSH, $\alpha$-subunit	Well differentiated cells. Weak or negative perinuclear LMWK.
Immature PIT1 lineage *		Monomorphic tumour cells; no hormones or one of more of: GH, PRL, $\beta$TSH, $\alpha$-subunit	Polygonal or chromophobic cells; cells lack features of terminal differentiation. Nuclear spheridia. Focal, variable LMWK.
Acidophil stem cell #		Monomorphic population of cells with predominant PRL and focal GH	Scattered fibrous bodies

IHC, immunohistochemistry; TF, transcription factor

* Newly defined in 2022 WHO Classification

# Newly described as separate “type” rather than “subtype” in 2022 WHO Classification

## Pit-1 plurihormonal tumours

**Fig. 1:**
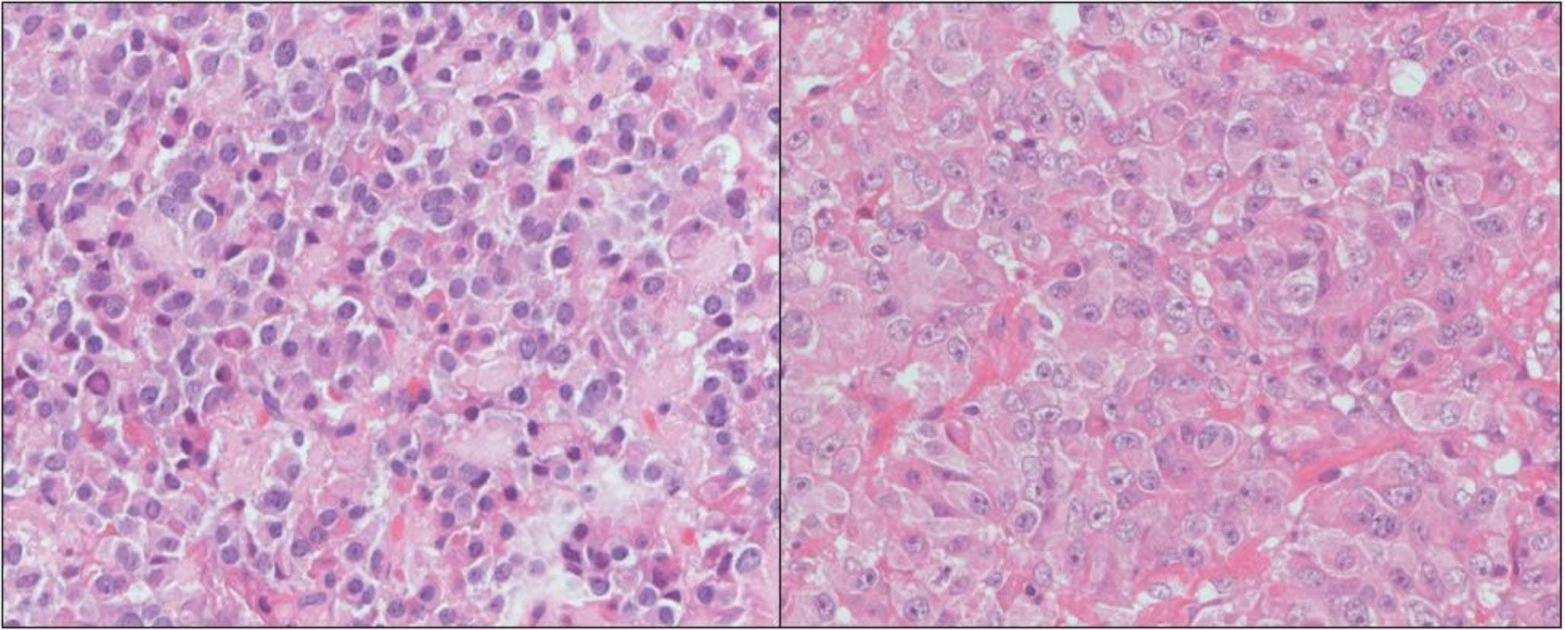
(a) Mature plurihormonal PIT1 lineage tumour (b) Immature PIT1 lineage tumour: large polygonal cells with marked nuclear atypia and macronucleoli

The nomenclature “Pit-1 positive plurihormonal tumour” was coined in the WHO 2017 classification as an alternative to the previously known “Silent subtype 3 adenoma”. However, the WHO 2022 edition refined this into 2 separate types being the “Immature Pit-1 lineage” (IPL) and “Mature plurihormonal Pit-1 lineage” (MPL) tumours to address the heterogeneity within the group. IPL (Fig. 1b) are characterised by chromophobic, polygonal tumour cells that lack features of terminal differentiation, with variable, if any, hormonal expression. The ultrastructural hallmark is the presence of nuclear spheridia. Conversely, MPL (Fig. 1a) are characterised by monomorphic, mature or well-differentiated tumour cells with predominant GH expression and frequently other Pit-1 hormone positivity such as PRL, TSH and/or α subunit, hence the name “plurihormonal” [1].

IPL better reflect tumours once described as “silent subtype 3” and are often associated with refractory behaviour. They are rare tumours accounting for just 0.9–1.5% of pituitary tumours, found more commonly among younger patients with a female preponderance [2, 3]. Hormonal hypersecretion occurs in only 30%, and may be associated with hyperthyroidism, acromegaly or hyperprolactinaemia [2]. IHC results vary, ranging from absent to multiple hormone positivity [2, 4]. Tumours are typically large with invasive disease in up to 60% and consequent low gross total resection rates in only 30–56% of patients [2–4]. Progression following incomplete resection is common, occurring in 53% of patients over 48 months [4]. Response to conventional radiation has been described, albeit in very small patient numbers [2–4]. Some tumours express somatostatin receptors (SSTR) and may respond to somatostatin analogue therapy (SSA)[3]. Low 06-methylguanine-DNA methyltransferase (MGMT) expression has been reported in 1 small study among 18/23 (78%) of “silent subtype 3” tumours which suggests potential efficacy of treatment with the alkylating agent temozolomide [5].

## Thyrotroph adenomas

Thyroid stimulating hormone (TSH)-secreting adenomas (thyrotroph adenomas (TA) or “TSHomas”) account for 2–3% of pituitary adenomas, although higher prevalence rates in the past 2 decades are attributed to a rise in detection of microadenomas [6–8]. TAs may present with isolated TSH elevation and consequent hyperthyroidism, but in around 40% of cases co-secretion of other hormones predominantly growth hormone (GH) and prolactin (PRL) (Fig. 1c,d) [8]. Plurihormonal expression, as assessed by IHC, maybe even more common, up to 83% in one study [7]. In fact, transcriptomic analysis demonstrates thyrotroph and plurihormonal Pit1 positive adenomas cluster with sparsely granulated somatotroph adenomas sharing a distinct gene expression profile different from lactotroph and somatotroph adenomas [9]. This suggests TA and Pit-1 plurihormonal tumours are more closely related than other tumours derived from the Pit-1 lineage. However, this study did not differentiate between mature or immature forms of Pit-1 plurihormonal tumours. TA, frequently presenting as large, invasive tumours, are typically considered aggressive, however the inclusion of IPL may account for this misrepresentation. In a recent review of 535 cases, plurihormonality was found to be more common among macroadenomas (51%) compared with microadenomas (27.3%)[10]. In fact, among a total cohort of 249 patients with aggressive pituitary tumours (APT) and pituitary carcinomas (PC) comprising both European Society of Endocrinology (ESE) surveys of 2016 and 2020, there were just 5 TA (2%), *none* of which were PC [11, 12]. Aggressive TA are also rarely encountered among other large case series of APT/PC, with just 3 published case reports of TSH-secreting PC [13–20]. In a recent French series of 20 TAs, there was just 1 tumour with Ki67 > 3% and none were classified as Grade 2b which are known to recur at a significantly higher rate, noting specifically that plurihormonal tumours included in their cohort were *not* of the “poorly differentiated” Pit-1 subtype[21, 22].

There appears to be a male predilection among aggressive TAs, with 2/3 PCs and all 5 across ESE surveys being male, compared with a 1.07 F:M ratio among all published TA cases [10]. This sex difference was also described in a large Chinese cohort of 111 TAs in which 10/12 co-secreting tumours were male compared with 58% women in the pure TA group, with co-secretors demonstrating significantly larger tumours with higher rates of cavernous sinus invasion[6]. Among the described aggressive TAs, including the 3 PC cases, there is a high proportion of “silent” TAs, which frequently become clinically functioning heralding more aggressive behaviour, and these may well represent IPL [11, 13–15]. This highlights the importance of detailed IHC analysis including Pit-1, ERα, GATA3 and low molecular weight cytokeratin to accurately distinguish mature TA from MPL and IPL. Whether truly “silent” pure TAs have a worse outcome remains unclear [8]. It has also been suggested that TAs may become more aggressive following thyroid ablation (similar to Nelson’s syndrome phenomena for corticotroph tumours) either from surgery or radioiodine that often results from incorrect diagnosis of TA as primary thyroid disease [23, 24]. In one PC case radioiodine thyroid ablation was administered because of poor compliance with antithyroid medication, with development of metastases 10 months subsequently[14]. However, while invasive macroadenomas have been described in this setting so have microadenomas, and in an NIH cohort there was no difference in tumour size between patients with a treated thyroid and those without [24–26]. Furthermore, there is often years before diagnosis of TA following thyroid ablation suggesting the natural history of tumour development may not have been perturbed. Somatic mutation of TRβ and aberrant expression of iodothyronine deiodinase enzyme expression have been linked with the resistance to thyroid hormone feedback of TSH regulation within TA but has not been associated with aggressive behaviour [27, 28]. In fact, little is known about the molecular mechanisms driving TA development – in a whole exome sequencing study of 8 TAs no recurrent mutations were found [29].

Transsphenoidal pituitary surgery remains first-line treatment for TAs as described in European Thyroid Association guidelines published in 2013 [30]. Overall among 535 reported cases, surgical remission rates are 69.7%, higher among microadenomas (87%) than macroadenomas (49%) [8]. Cavernous sinus invasion is the strongest predictor of surgical outcome with 75% of Knosp Grade 3 versus 0% Knosp Grade 4 tumours achieving remission in 1 modern study [7]. Preoperative use of SSA therapy does not appear to improve remission rates, although may be used to prevent peri-operative thyroid storm [8, 31]. Recurrence following gross total resection is uncommon in the first 3 years particularly if there is a low TSH in the 1st week postoperatively [7, 21, 32]. Further surgery for recurrent disease is associated with lower gross total resection rates (28.57% versus 71.42% primary surgery in 1 study) [33]. In cases not achieving remission or with recurrence, SSA treatment is effective and should be considered first-line medical therapy following incomplete surgery. In a meta-analysis of 536 TAs biochemical remission was seen in 76% of cases under SSA therapy with other cohorts demonstrating significant tumour shrinkage in up to 50%, but just an isolated case of complete remission [8, 23]. SSTR5 expression may predict long term response to SSA therapy with one case of aggressive behaviour developing in the context of LOH involving the SSTR5 gene [34–36]. Radiotherapy (RT) may be used as second-line therapy, but now more frequently in setting of SSA resistance or concern about long-term SSA with total thyroidectomy only indicated for life-threatening hyperthyroidism when pituitary surgery not curative [10]. In a study of 19 macroTAs, biochemical remission was seen in 21% up to 2 years after RT with 37% still on medical therapy at last followup [31]. All patients in whom tumour shrinkage was evident received radiosurgery (rather than fractionated RT) with complete remission in 1 patient. In those resistant to SSA, there may be utility in trialling dopamine agonist therapy with a few cases demonstrating response but also occasional paradoxical increases in TSH have been seen [25, 37]. Efficacy of temozolomide therapy in the setting of progressive disease despite SSA and RT in TA remains unclear based on limited cases. Of 6 published cases (5 APT, 1 PC), noting 5 of these were “silent”, there was just 1 case of partial remission (41% tumour reduction) with 3 demonstrating stable disease and the PC progressing [11, 15, 38]. In 3 where MGMT IHC was performed, low expression was seen in one case of stable disease and intermediate expression in a case with partial response and another with stable disease. As commonly seen in historical cohorts of APT/PC, other chemotherapy regimes have been unsuccessful [13]. Use of PRRT, immunotherapy or targeted molecular therapies such as bevacizumab (VEGF inhibitor) in TA have not yet been described, but given occasional benefit in other APT/PC are of interest.

## Conclusion

Pit-1 plurihormonal tumours comprise both mature (MPL) and immature (IPL) types now recognised in the WHO 2022 classification. These tumours have gene expression profiles that are closely aligned with TA, although IPL more closely resembles the previously known silent subtype 3 adenoma and may account for the poorer prognosis often attributed to these Pit-1 lineage tumours. In cases with residual or recurrent disease following surgery, a trial of SSA is warranted and RT may be effective. Low MGMT expression may be seen more frequently in IPL but data on temozolomide efficacy is limited but should be first-line chemotherapy in the absence of other known effective therapies.

## Data Availability

Not applicable.
